# Neutrophil Gelatinase-Associated Lipocalin (NGAL) Is Related with the Proteinuria Degree and the Microscopic Kidney Findings in *Leishmania*-Infected Dogs

**DOI:** 10.3390/microorganisms8121966

**Published:** 2020-12-11

**Authors:** María Paz Peris, Mariano Morales, Sonia Ares-Gómez, Adriana Esteban-Gil, Pablo Gómez-Ochoa, Manuel Gascón, Bernardino Moreno, Juan Antonio Castillo

**Affiliations:** 1 Veterinary Faculty, AgriFood Institute of Aragon-IA2, University of Zaragoza-CITA, 50013 Zaragoza, Spain; mjma1962@gmail.com (M.M.); adriesgil@gmail.com (A.E.-G.); pablogomezochoa@gmail.com (P.G.-O.); manuelgp@unizar.es (M.G.); bmoreno@unizar.es (B.M.); jacasti@unizar.es (J.A.C.); 2 ISGlobal, Hospital Clínic-University of Barcelona, 08036 Barcelona, Spain; soniaares@gmail.com

**Keywords:** canine leishmaniasis, NGAL, kidney disease, proteinuria, glomerular lesions

## Abstract

Early diagnosis of renal damage in *Leishmania* infected dogs may allow appropriate treatments and prevent some deaths. This study investigates neutrophil gelatinase-associated lipocalin (NGAL) as a biomarker of kidney disease in dogs experimentally infected with *Leishmania infantum*. Serum, urine, and kidney samples were collected from 30 infected beagle dogs and six uninfected control dogs. Based on proteinuria and azotemia values, dogs were initially classified. NGAL was measured in urine and serum samples. Then, the urinary NGAL to creatinine ratio (uNGAL/C) was calculated. Kidney samples were taken for histopathological studies, and the dogs were classified according to the severity of glomerular and tubulointerstitial lesions. In *Leishmania*-infected dogs, the uNGAL/C was significantly higher in proteinuric non-azotemic dogs compared with non-proteinuric non-azotemic dogs (*p* = 0.038). Serum NGAL (sNGAL) concentration did not differ between groups. Microscopic studies revealed several degrees of glomerulonephritis and slight focal lymphoplasmacytic interstitial nephritis in 89% and 55% of infected dogs, respectively. Urinary protein to creatinine ratio (UPC) and uNGAL/C were significantly higher in dogs with affected glomeruli compared to infected dogs without renal lesions (*p* = 0.045 and *p* = 0.043, respectively). The results show that uNGAL/C correlates with proteinuria and the presence of moderate glomerular lesions in non-azotemic dogs experimentally infected with *L. infantum*.

## 1. Introduction

Canine visceral leishmaniasis is caused by the protozoan *Leishmania infantum*, which affects several organs, for example, the skin, kidneys, spleen, liver, and eyes, and is characterized by a range of associated clinical signs [1]. Renal lesions are characterized by glomerular and interstitial lesions, typically associated with immune complex deposition [2]. Consequently, *Leishmania*-infected dogs may develop acute kidney injury (AKI) or, more frequently, chronic kidney disease (CKD) [3,4]. Diagnosing and treating kidney injury in its early stages is crucial for minimizing and potentially avoiding chronic and fatal progression of kidney disease [1].

Current protocols for the treatment of canine leishmaniasis are mainly based on the renal status, which is estimated following the International Renal Interest Society guidelines (IRIS Group) [5,6]. They recommend measuring serum creatinine (sCr) concentration and the UPC for diagnosing, staging, and treating both AKI and CKD. The UPC quantitatively measures proteinuria and is routinely used in canine leishmaniasis diagnosis, due to its increase being one of the first abnormalities occurring in renal impairment [6,7]. However, sCr, a kidney function marker, is insensitive for diagnosing an early renal injury, before functional impairment occurs, and it is only detected when at least 75% of nephrons are non-functional [8]. Although histopathology is the best method to assess early renal damage for studying proteinuric kidney diseases [9], a kidney biopsy is an invasive technique and not routinely performed. Ultrastructural studies are typically required for an adequate evaluation of renal damage, but light microscopy is more often performed for studying proteinuric kidney diseases, mainly due to cost and technical reasons [9]. Consequently, recent studies assert the necessity of more sensitive biomarkers of early damage in canine renal diseases [10], and alternative urinary biomarkers of renal damage are increasingly studied. A number of these biomarkers can localize the site of the renal injury. For example, immunoglobulin G (IgG) [11], C-reactive protein (CRP) [12], and ferritin [13] concentrations in urine are associated with glomerular lesions, whereas *N*-acetyl-β-d-glucosaminidase (NAG) [14] or gamma glutamyl-transpeptidase (GGT) [15,16] levels reflect tubular lesions. These biomarkers have been compared with traditional tests; however, they have been infrequently correlated with histopathological lesions in kidneys [16,17].

Neutrophil gelatinase-associated lipocalin (NGAL) is a glycoprotein with a molecular weight of 25 kDa and a member of the lipocalin family. It was initially purified from neutrophils during infection and inflammation [18]. However, NGAL is also expressed in the uterus, prostate gland, salivary glands, bone marrow, stomach, colon, trachea, lungs, liver, and kidneys [19]. NGAL is considered a good biomarker for both the early diagnosis of renal injury and the evaluation of its progression [20]. Moreover, it has been shown to predict adverse outcomes of CKD, such as end-stage renal disease [21,22,23]. Additionally, NGAL has been associated with several conditions in humans, including immunological [24], metabolic (i.e., diabetes) [25], and inflammatory (i.e., acute generalized peritonitis) [26] diseases.

It is one of the most studied biomarkers in veterinary medicine [27] and may be a good early biomarker of both acute and chronic kidney injury [9,27,28,29]. In dogs, increasing evidence shows that urinary NGAL (uNGAL) correlates with the degree of renal damage [10]. It seems to be a sensitive and specific marker of AKI, although it remains to be determined whether NGAL is an early predictor of CKD [30]. However, it has not been studied in dogs with leishmaniasis.

As kidney disease is frequent in *Leishmania*-infected dogs and may lead to end-stage renal failure [31], the aim of the study was to investigate uNGAL values in dogs experimentally infected with *L. infantum* and compare them with the renal parameters routinely used (sCr and UPC values) and with the microscopic findings observed in the kidney. This work shows that uNGAL increases in proteinuric dogs without azotemia, and that correlates with the presence of glomerular lesions observed by histopathology in dogs experimentally infected with *L. infantum*.

## 2. Materials and Methods

### 2.1. Animals, Samples, and Ethical Statements

Thirty (15 females and 15 males) intact, healthy, eight-month-old, ~13 kg beagle dogs were experimentally infected intravenously with one mL of an inoculum that had a concentration of 1 × 10^8^ promastigotes/mL of *L. infantum* (isolate MCAN/ES/Z002) obtained as previously described [32]. The isolate corresponded with genotype A (Maribel Jiménez and Ricardo Molina, personal communication) according to ITS-based genotyping [33]. Additionally, six beagles (three females and three males) were kept as uninfected controls. All these dogs were a subsample from a larger experiment in which all animals were periodically examined to determine their health status: ~180 days post-infection (dpi), ~240 dpi, ~300 dpi, and ~360 dpi. Special consideration was given to clinical renal status, and no medical treatments that could affect kidneys were administered to any dog throughout the experiment. All animals received regular exercise and social interaction and were housed, maintained, and used for experimentation at optimal conditions: Ad-libitum feeding, procedure refinement, disease monitoring, behavioral enrichment, and temperature control.

At the end of the study, ~360 dpi, animals were euthanized following sedation, by using 0.3 mL/kg of T61 (Intervet) by the intravenous route. Afterward, a complete necropsy was performed. Prior to euthanasia, blood and urine samples were taken. Blood samples were obtained by cephalic vein puncture and urine samples by guided ultrasound cystocentesis.

One mL of urine was used to perform a complete urinalysis and evaluation of the sediment. Pyuria was considered when more than 10 leukocytes/µL or more than six altered leukocytes per high-power field (400×) were observed. No abnormal results were found. The remaining urine was centrifuged at 300× *g* for two minutes and kept at −80 °C for one month until NGAL analysis. Blood samples were centrifuged at 1200× *g* for ten minutes to obtain the serum and kept at −20 °C for further studies. One dog was not sampled because had to be euthanized before the end of the experiment (after the ~240 dpi time point). The animal reached the animal welfare endpoints set before the study.

All applicable international, national, and/or institutional guidelines for the care and use of animals were followed (Spanish Policy for Animal Protection RD53/2013, which meets the European Union Directive 2010/63 on the protection of animals used for experimental and other scientific purposes). All efforts were made to minimize suffering. All experimental practices involving animals were approved by the Ethics Committee for Animal Experiments from the University of Zaragoza (Project license PI28/14, date of approval: 4 June 2014).

### 2.2. Clinical Signs and Serological Study

Clinical signs evaluation and serological determinations to confirm the infection was carried out every two months from 180 dpi. Until the end of the experiment (~180 dpi, ~240 dpi, ~300 dpi, and ~360 dpi). Clinical signs characteristics of leishmaniosis were specially assessed: Weight loss, peripheral lymphadenomegaly, skin lesions (desquamative dermatitis, alopecia, and ulcers), temporal muscle atrophy, splenomegaly, polyuria/polydipsia, epistaxis, ocular lesions, onychogryphosis, lameness, and vomiting/diarrhea. Dogs without signs were considered asymptomatic, dogs with less than three signs were considered oligosymptomatic, and dogs with three or more clinical signs were classified as polysymptomatic. Additionally, clinical signs related with urinary tract infections (UTI), such as stranguria, pollakiuria or dysuria, were also assessed.

*Leishmania* serology was done using two techniques. A commercial ELISA kit with a sensitivity of 92.5% and a specificity of 100% [34] (CIVTEST CANIS LEISHMANIA 192, Hipra Laboratories S.A. Gerona, Spain). The technique was performed according to the manufacturer’s instructions and the sera were considered negative (Rz < 0.9), doubtful (0.9 < Rz < 1.1), low positive (1.1 < Rz < 1.5) and positive (Rz > 1.5). On the other hand, a direct agglutination test (DAT) was used as previously reported [35], with a sensitivity of 100% and a specificity of 98.7%. Negative results were considered when DAT dilution was <1/200, doubtful when dilution was 1/400, low positive when dilution was 1/800, and positive when dilution was >1/800.

### 2.3. Biochemical and Hematological Analyses

At ~180 dpi, ~240 dpi, ~300 dpi, and ~360 dpi, biochemical and hematological analyses were carried out. Biochemical determinations were performed for albumin (ALB), alkaline phosphatase (ALP), Urea, Creatinine (sCr), aspartate aminotransferase (AST), alanine aminotransferase (ALT), lactate dehydrogenase (LDH), total bilirubin (T-Bil) and total protein (T-Pro) with the Automatic Analyser Gernon Star (RAL, Barcelona, Spain). Routine hematological analyses were also performed to assess anemia, leukopenia, and thrombocytopenia with the hematological equipment XT-SYSMEX 2000 iV (Roche Diagnostics, Barcelona, Spain).

### 2.4. Proteinuria and Azotemia Determinations and Group Allocation

To assess proteinuria, during the last month, three urine samples were collected every 15 days. The UPC was calculated to estimate the degree of proteinuria. Urinary total proteins were measured by the pyrogallol red-molybdate method. Creatinine levels in serum and urine were measured by the creatinase and sarcosine enzymatic method (Automatic Analyser Gernon Star, RAL, Barcelona, Spain).

Dogs were classified according to UPC and sCr values based on the guidelines of the IRIS Group [36]. Depending on UPC values, dogs were considered as non-proteinuric (UPC < 0.2), borderline proteinuric (UPC between 0.2–0.5) and proteinuric (UPC > 0.5). Azotemia was defined as sCr > 1.4 mg/dL.

To assess if NGAL was sensitive enough to detect renal disease, dogs were classified into four groups using both sCr and UPC: Group 1, non-proteinuric and non-azotemic dogs; Group 2, borderline proteinuric and non-azotemic dogs; Group 3, proteinuric and non-azotemic dogs; Group 4, proteinuric and azotemic dogs [13].

### 2.5. Serum NGAL, Urinary NGAL, and uNGAL/C Determinations

As NGAL can also increase with inflammatory conditions [24], and inflammation is typically observed in *Leishmania*-infected dogs, NGAL was also determined in serum to discard an inflammatory origin. Furthermore, it has been suggested that uNGAL and sNGAL should be evaluated together to minimize any influence from other systemic diseases [37]. Serum and urine samples collected at ~360 dpi were thawed to realize the NGAL determinations. Serum and urine NGAL concentrations were measured with a commercial ELISA kit validated in dogs (Dog NGAL ELISA Kit, Bioporto Diagnostics A/S, Gentofte, Denmark) following the manufacturer´s instructions. The absorbance was measured with an ELISA reader (Tecan Sunrise Microplate Reader, Switzerland) at 450 nm. For statistical analysis, values below the detection limit (4 pg/mL) were assigned a value of 2 pg/mL [38]. The urinary concentration of NGAL may be influenced by hydration status or urinary concentration capacity. Accordingly, the uNGAL/C was used to control for individual variations, as it adjusts for heterogeneity in urinary concentration [38]. For urine samples, intra- and inter-assay coefficients of variations (CV) were below 2% and 9%, respectively, and for plasma samples, they were below 3% and 8%, respectively. Then, sNGAL and uNGAL concentrations were expressed as mg/dL.

### 2.6. Histopathological Study and Group Allocation

To assess the condition of the nephrons and the origin of the damage, a histopathological study was performed in 27 infected and six uninfected dogs (three infected dogs were not sampled, due to protocol reasons). Kidneys were evaluated for gross lesions and samples were taken for microscopic studies. Samples were routinely processed. Briefly, samples were fixed in buffered formalin, embedded in paraffin, and cut at 4 µm. Two sections per kidney were obtained, and cortical, medullar, and pelvis areas were examined. Sections were stained by Hematoxylin and Eosin (H-E) and Periodic Acid-Schiff (PAS).

Glomerular lesions were evaluated in all animals according to the recent consensus of the World Small Animal Veterinary Association Renal Pathology Initiative [9,10]. Lesions typically associated with leishmaniasis, such as membranoproliferative glomerulonephritis (MPGN) or mesangioproliferative glomerulonephritis (PGN) and glomerulosclerosis were assessed. Such lesions were evaluated in 20 random high-power fields (400×) in each kidney. The number of affected glomeruli, as well as the extension of the lesion within each glomerulus (segmental or diffuse), was used to classify the dogs into three groups [2]: Group A, dogs with no glomerular alterations; Group B, dogs with <50% of glomeruli affected; and Group C, dogs with ≥50% of glomeruli affected. Tubular and interstitial lesions were also considered, and the damage of the tubular epithelium, presence of fibrosis, and characteristics (a type of inflammatory cells and extension) of the interstitial inflammation were evaluated as previously described [10], with some modifications. The resulting groups consisted of: Group I, dogs with no interstitial inflammation; Group II, dogs with mild, focal, interstitial inflammation; Group III, dogs with moderate, focal to multifocal, interstitial inflammation; and Group IV, dogs with severe, diffuse, interstitial inflammation.

### 2.7. Statistical Analysis

The Shapiro-Wilk test was performed to assess the normality of data. For non-normally-distributed data, the non-parametric Kruskal-Wallis test was used to determine any statistically significant differences between groups. Dunn´s post hoc test adjusted by Bonferroni was used for pair-wise group comparison. Correlations between the parameters studied were determined using Spearman correlation analysis. Statistical analyses were performed with SPSS v24 (IBM Corporation, Armonk, NY, USA), and statistical significance was set at α = 0.05.

## 3. Results

### 3.1. Clinical Signs and Laboratory Studies

Infected dogs showed clinical signs from ~240 dpi, and 20 were classified as oligosymptomatic and nine as polysymptomatic. Signs increased throughout the experiment, and ~360 dpi three animals were classified as oligosymptomatic and 26 as polysymptomatic. Dogs showed doubtful or low positive antibody levels against *Leishmania* at ~180 dpi (21 and eight dogs, respectively); however, at ~240 dpi and onwards throughout the study, all infected animals were seropositive. Regarding biochemical results (Table 1), only LDH, urea, and sCr were altered. LDH was increased in five animals from ~240 dpi and onwards and urea in three animals at ~360 dpi. Serum creatinine increased more than 0.2 mg/dL between ~240 dpi and ~300 dpi in 25 animals. These values remained constant until the end of the study; however, no azotemia values (sCr > 1.4 mg/dL) were reached in any dog. Hematological studies revealed no anemia, leukopenia, or thrombocytopenia in any dog throughout the study. Negative control dogs presented no antibodies, laboratory abnormalities, or clinical signs compatible with leishmaniasis.

### 3.2. NGAL Values and Proteinuria Degree

**Figure 1:**
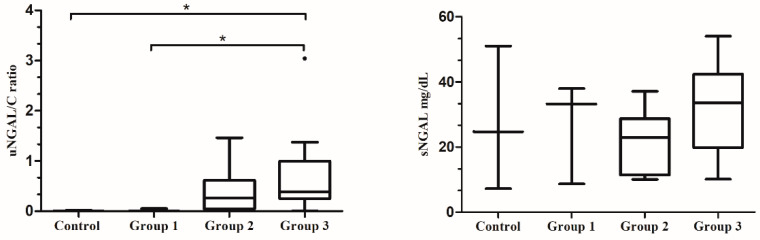
Box plots showing the relationship between uNGAL/C and sNGAL and the different groups of proteinuria degree. Data are presented as boxes and whiskers. Each box includes the 25 and 75 interquartile, whereas the line inside the box represents the median, the whiskers represent the minimum and maximum values. The outlier is shown by a circle. Asterisks indicated significant differences between groups: * *p* < 0.05.

Regard to renal parameters and based on the classification used in this study for UPC and sCr parameters, Group 1 included three dogs, Group 2 included eight dogs, Group 3 included 18 dogs, and none were classified as Group 4. Statistical analyses showed that uNGAL/C medians differed significantly among groups (*p* = 0.004). Post-hoc comparisons showed that this ratio differed significantly between the control group (median, 0.003; range, 0.002 to 0.018) and Group 3 (median, 0.039; range, 0.006 to 3.043) (*p* = 0.032), and between the Group 1 (median, 0.003; range, 0.002 to 0.51) and Group 3 (median, 0.039; range, 0.006 to 3.043) (*p* = 0.038). In addition, uNGAL/C median values increased from Group 1 to 3. However, no significant differences were found in sNGAL determinations between the control group (median, 24.73 mg/dL; range, 7.24 mg/dL to 51 mg/dL), Group 1 (median, 33.22 mg/dL; range, 8.67 mg/dL to 37.96 mg/dL), Group 2 (median, 22.86 mg/dL; range, 10.08 mg/dL to 37.14 mg/dL), and Group 3 (median, 0.39 mg/dL; range, 0.006 mg/dL to 3.043 mg/dL) (*p* = 0.387), and there was no increase between Groups 1 to 3 (Figure 1).

**Figure 2:**
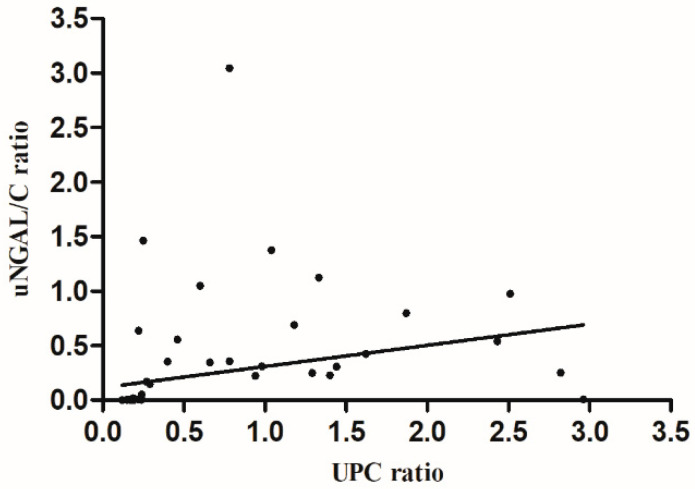
Graphic representation of Spearman correlation between uNGAL/C and UPC (rs = 0.485, *p* = 0.005).

The Spearman correlation test did not reveal a statistically significant positive correlation between sNGAL and sCr (rs = 0.267, *p* = 0.14), nor between sNGAL and uNGAL (rs = 0.33, *p* = 0.062). However, the UPC and uNGAL/C showed a statistically significant positive moderate correlation [39] (rs = 0.482, *p* = 0.005) (Figure 2).

### 3.3. NGAL Values and Histopathological Study

None of the experimentally infected dogs presented gross lesions in the kidneys; however, 24 (89%) of them showed microscopic lesions. No histological changes were observed in three (11%) infected animals or in control dogs.

**Figure 3:**
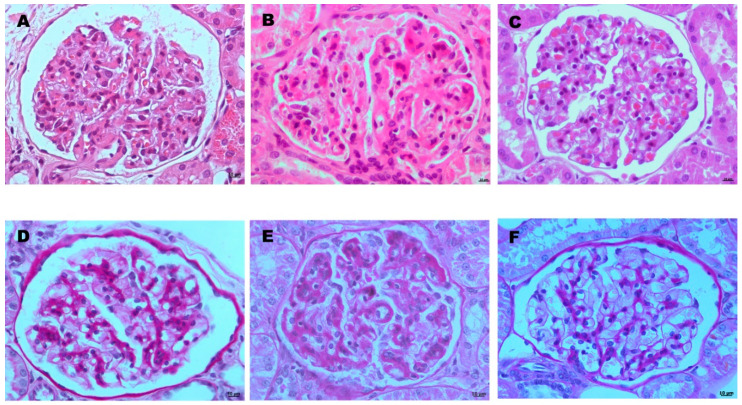
Representative microphotographs of glomerular lesions in dogs experimentally infected with *Leishmania infantum* (**A**,**B**,**D**,**E**). (**C**,**F**) correspond with control dogs showing normal glomeruli. (**A**–**C**) are stained with Hematoxylin and Eosin (H-E) and (**D**–**F**) with Periodic Acid-Schiff (PAS), and all have an original magnification of 400×. (**A**,**D**): Mesangioproliferative glomerulonephritis. Note groups of three mesangial cells (**A**), hallmark of this type of glomerulonephritis in dogs and moderate increase in mesangial tissue (**D**). (**B**,**E**): Membranoproliferative glomerulonephritis. Note the marked increase in basal membranes, best observed with PAS staining (**E**). A mild increase in cellularity is observed in some areas (**E**).

Glomerulonephritis was observed in 24 infected dogs, including MPGN (11/24 dogs; 46%) and PGN (13/24 dogs; 54%) (Figure 3). According to the number of affected glomeruli, 13 dogs had less than 50% of glomeruli injured (Group B), while 11 had more than 50% affected (Group C).

**Figure 4:**
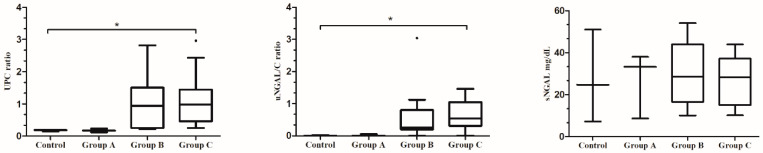
Box plots showing the relationship between UPC, uNGAL/C, and sNGAL and the different groups of dogs according to the glomerular lesion’s classification. Data are presented as boxes and whiskers. Each box includes the 25 and 75 interquartile, whereas the line inside the box represents the median, the whiskers represent the minimum and maximum values. Outliers are shown by a circle. Asterisks indicated significant differences between groups: * *p* < 0.05.

UPC and uNGAL/C medians differed significantly among groups (*p* = 0.004 and *p* = 0.006, respectively. Post-hoc comparisons showed that UPC differed significantly between the control group (median, 0.19; range, 0.15 to 0.19) and Group C (median, 0.98; range, 0.25 to 2.96) (*p* = 0.045). Similarly, the uNGAL/C differed significantly between the control group (median, 0.003; range, 0.002 to 0.018) and Group C (median, 0.54; range, 0.006 to 1.462) (*p* = 0.043). In terms of the data distribution (range values), UPC showed higher variability in each group than uNGAL/C values. In addition, uNGAL/C median values increased from Groups A to C, while UPC showed no clear increase between Groups B and C. On the other hand, when NGAL was determined in serum, no significant differences were found between control group (median, 24.73 mg/dL; range, 7.24 mg/dL to 51 mg/dL), Group A (median, 33.22 mg/dL; range, 8.67 mg/dL to 37.96 mg/dL), Group B (median, 28.63 mg/dL; 10.08 mg/dL to 54.06 mg/dL) and Group C (median, 28.18 mg/dL; range, 10.10 mg/dL to 43.9 mg/dL) (*p* = 0.96) and increase was not observed between Groups A to C (Figure 4).

Interstitial lesions were seen in 15 of 27 infected dogs (55%) and were characterized by small foci of lymphoplasmacytic interstitial nephritis, mainly located in the corticomedullary junction and renal pelvis. All these dogs were included within Group II. According to interstitial inflammation, UPC, uNGAL/C, and sNGAL values did not show statistical differences between dogs with interstitial nephritis and animals without inflammatory changes (Group I) (*p* = 0.078, *p* = 0.06, and *p* = 0.79, respectively). No tubular lesions were observed in the animals.

## 4. Discussion

To the author´s knowledge, this is the first study of uNGAL and sNGAL estimations in dogs experimentally infected with *L. infantum*. This study has shown that uNGAL/C is higher in non-azotemic *Leishmania*-infected dogs with proteinuria compared with non-proteinuric dogs. This finding suggests that uNGAL may detect mild glomerular lesions with proteinuria. Interestingly, uNGAL has also been considered a good marker of renal recovery during specific treatments in humans with low-level proteinuria [40]. Similar studies in canine leishmaniasis would be beneficial. However, uNGAL does not seem to predict deaths in azotemic dogs when CKD is clinically evident or azotemic AKI dogs, in contrast with sCr [27,41]. In our study no azotemic dogs were found, avoiding estimations of these parameters.

A major limitation of NGAL studies is the difficulty to determine whether uNGAL increases exclusively in response to kidney damage, or it is due to other conditions [29]: Increasing glomerular NGAL filtration in otherwise normal glomeruli might occur in systemic conditions, such as immunological, metabolic, and inflammatory diseases, due to an increased sNGAL [24,37]. In this study, NGAL was also determined in serum to rule out the role of systemic diseases. An important advantage of our experimental protocol is that it allowed us to exclude most of these diseases. No significant increase of sNGAL compared with the control group was detected, suggesting that the increase in uNGAL was of renal origin and not from systemic inflammation. Although evaluation of inflammatory markers would have been needed to assess the systemic inflammation status, a mild systemic inflammation was observed after the histopathological study of all organs in all infected dogs (these data are not shown because they are part of other study). Thus, we consider that, under our experimental conditions, *Leishmania* was able to induce mild inflammation; however, this degree of inflammation is not enough to induce an increase in sNGAL. Further studies, however, are needed in natural cases and dogs with different degrees of systemic inflammation, due to *Leishmania* infection to estimate possible variations of NGAL in serum.

In addition to increasing glomerular filtration, deficiency of tubular reabsorption and tubular production can also influence uNGAL concentration [40]. Urinary NGAL has been associated with tubular or tubulointerstitial injuries [9,10,28]. Furthermore, when tubulointerstitial lesions are severe as in end-stage kidneys, uNGAL may also be increased as a result of glomerular proteinuria [10]. Our study revealed that after one year of the infection with *L. infantum*, renal lesions were predominantly glomerular with insignificant tubulointerstitial changes. Therefore, it can be speculated that uNGAL mainly reflects glomerular damage in the absence of tubular injuries. Both UPC and uNGAL/C significantly increased in animals, with more than 50% of glomeruli affected (Group C). However, UPC values were broadly dispersed compared with uNGAL/C, suggesting that uNGAL is a more robust biomarker.

In contrast, although no significant tubulointerstitial lesions were observed in the present study, and uNGAL was suspected to be associated with glomerular lesions, tubular participation cannot be discarded. Besides to tubular degeneration, insufficient tubular reabsorption may also be due to saturation of its absorptive capacity. Albumin is a protein with low molecular weight and is considered to be the first protein detected in cases of proteinuria [42]. Because NGAL is smaller than albumin, they are expected to be filtrated together. As both are reabsorbed by the same receptors [40], competition might occur. This situation is expected in low-level proteinuria and may occur in the early stages of kidney disease; however, in these cases, creatinine has not been clearly shown to increase, so uNGAL estimation could detect earlier kidney changes.

On the other hand, NGAL production by tubular cells as a protective function has also been demonstrated [40], and this situation is thought to occur in mild renal injuries. Reabsorption for saturation and greater production in early renal pathology might explain the greater sensitivity of the uNGAL/C when compared to UPC in detecting mild renal lesions. This possible explanation would be in accordance with the Forest Fire theory. In this analogy, nephrons represent the trees in the forest, and NGAL represents the fire. After damage, the remaining nephrons re-establish renal function, which is commonly evaluated as the sCr concentration. In this model, NGAL indicates the active lesions during renal damage [43]. This theory aligns well with our histological findings, in which no tubulointerstitial lesions were detected, and only glomerular lesions were seen. In this situation, tubular cells were able to produce NGAL, in contrast with tubular cells seen in chronic-end-stage kidneys where interstitial lesions predominate. This condition typically occurs in natural cases of canine leishmaniasis when the disease is diagnosed and presents a very poor prognosis [2].

Renal lesions in dogs infected by *L. infantum* are characterized by glomerular and tubulointerstitial changes [2]. Proteinuria, due to glomerular lesions and tubular saturation, has been suggested as a contributing factor in the development of tubulointerstitial injuries [21]. In this study, significant lesions were observed only in the glomeruli, whereas interstitial lesions, characterized by focal mild mononuclear interstitial nephritis, were insignificant. Moreover, interstitial lesions were not seen in all dogs with glomerular lesions. The post-infection time at which dogs were euthanized probably explained the lack of significant tubulointerstitial lesions. Nevertheless, statistical analyses comparing interstitial lesions and uNGAL/C values were performed, and no significant differences were detected (*p* = 0.06).

In our study, over 11% of the infected dogs showed no renal pathology. Glomerular lesions in canine leishmaniasis are characterized by the presence of MPGN, PGN, or glomerulosclerosis and are typically associated with immune-complex deposition [2]. In our study, no lesions of glomerulosclerosis were observed, suggesting that these chronic lesions are not yet developed after one year of infection. Both types of glomerulonephritis were seen, although PGN predominated over MPGN (54% and 46%, respectively). These percentages are similar to those reported by other authors [44]. The study confirms that *L. infantum* induces renal disease in dogs, in which glomerular damage is initially observed and that the response to infection varies between dogs, even within the same breed.

This work is an experimental study and has some limitations. The two main limitations have been the lack of azotemic dogs and the lack of some previous uNGAL determinations throughout the experiment. These data might have provided more relevant information about the potential value of this renal biomarker. Further studies are needed to verify these findings in natural infections by *L. infantum*. Another limitation of this experimental study was that UTI was not ruled out. Urinary tract infections and noninfectious pyuria have been suggested to influence uNGAL values [45,46]. Although no signs suggesting UTI were observed in our study such as pathological findings in the sediment (hematuria, pyuria), clinical signs (stranguria, pollakiuria, dysuria), urine culture would have been the only method to rule out the presence certain pathogens that can provoke asymptomatic infections.

## 5. Conclusions

This study demonstrates that uNGAL is increased in proteinuric and non-azotemic dogs, and that correlates with the presence of glomerular lesions in dogs experimentally infected with *L. infantum*. These results also suggest that sNGAL may not be a sensitive indicator of the levels of proteinuria.

## Figures and Tables

**Table 1:** Biochemical results at the sampling points. Number and mean ± SD values of animals with normal and altered values.

			ALB gr/dL	ALP IU/L	Urea mg/dL	sCr mg/dL	AST IU/L	ALT IU/L	LDH IU/L	T-Bil mg/dL	T-Pro gr/dL
180 dpi	Normal	N	29	29	29	29	29	29	29	29	29
		Value	4 ±0.15	59.9 ±34.3	35.5 ±5.3	0.67 ±0.1	25.7 ±5.5	41.3 ±8.6	117.5 ±40.7	0.39 ±0.06	6.25 ±0.4
	Altered	N	-	-	-	-	-	-	-	-	-
		Value	-	-	-	-	-	-	-	-	-
240 dpi	Normal	N	29	29	29	29	29	29	24	29	29
		Value	3.65 ±0.23	66.5 ±36.4	38.2 ±1.1	0.73 ±0.15	35.8 ±14.5	72.2 ±17	172.2 ±17.2	0.38 ±0.07	6.16 ±0.8
	Altered	N	-	-	-	-	-	-	5	-	-
		Value	-	-	-	-	-	-	303.1 ±15.2	-	-
300 dpi	Normal	N	29	29	29	4	29	29	24	29	29
		Value	3.72 ±0.28	57.5 ±30.3	32.3 ±6.2	0.72 ±0.12	42.6 ±19.6	69.3 ±15.7	185.5 ±24.8	0.37 ±0.03	6.84 ±0.6
	Altered	N	-	-	-	25	-	-	5	-	-
		Value	-	-	-	1.05 * ±0.09	-	-	358.8 ±21.7	-	-
360 dpi	Normal	N	29	29	26	4	29	29	24	29	29
		Value	3.6 ±0.32	77.3 ±50.9	33.1 ±5.6	0.73 ±0.08	49.1 ±7.6	78.4 ±22.3	191.4 ±12.5	0.36 ±0.05	6.73 ±1.03
	Altered	N	-	-	3	25	-	-	5	-	-
		Value	-	-	86.6 ±38.9	1.02 * ±0.04	-	-	683.2 ±257.9	-	-

* Increase of more than 0.2 mg/dL compared with 240 dpi. No animal reached azotemia values. Reference values: ALB: 2.3–4.6 gr/dL; ALP: < 212 IU/L; Urea: 20–40 mg/dL; sCr: 0.5–1.2 mg/dL; AST: < 60 IU/L; ALT: < 100 IU/L; LDH: 24–219 IU/L; T-Bil: 0.1–0.6 mg/dL; T-Pro: 5.7–7.5 g/dL.
